# Introduced Predator Elicits Deficient Brood Defence Behaviour in a Crater Lake Fish

**DOI:** 10.1371/journal.pone.0030064

**Published:** 2012-01-09

**Authors:** Topi K. Lehtonen, Jeffrey K. McCrary, Axel Meyer

**Affiliations:** 1 Lehrstuhl für Zoologie und Evolutionsbiologie, Department of Biology, University of Konstanz, Konstanz, Germany; 2 Section of Ecology, Department of Biology, University of Turku, Turku, Finland; 3 Estación Biológica, FUNDECI/GAIA, Laguna de Apoyo Nature Reserve, Nicaragua; Ecole Normale Supérieure de Lyon, France

## Abstract

Introduced species represent one of the most serious global threats to biodiversity. In this field-based study, we assessed behavioural responses of brood tending cichlid fish to an invasive predator of their offspring. This was achieved by comparing parental defence responses of the endangered arrow cichlid (*Amphilophus zaliosus*), a fish species endemic to the crater lake Apoyo in Nicaragua, towards the bigmouth sleeper (*Gobiomorus dormitor*), a formidable predator of cichlid fry, and all other potential fish predators of offspring. The bigmouth sleeper was recently introduced into Apoyo but naturally co-exists with cichlids in a few other Nicaraguan lakes. Arrow cichlid parents allowed bigmouth sleepers to advance much closer to their fry than other predators before initiating aggressive brood defence behaviours. Interestingly, parents of a very closely related species, *A. sagittae*, which has coevolved with bigmouth sleepers in crater lake Xiloá, reacted to approaching bigmouth sleepers at comparable distances as to other predators of cichlid fry. These results provide a novel demonstration of the specific mechanism (i.e. naive parental behaviour) by which invasive predators may negatively affect species that lack the adequate behavioural repertoire.

## Introduction

Introduced species that have subsequently become abundant in their new habitats (often called ‘invasive species’) are considered to be one the leading global threats to biodiversity [1], [2], with ample evidence suggesting that freshwater ecosystems may be especially susceptible to the influence of species introductions [3]–[5]. This pattern may be due to lack of coevolution among prey and novel predators in many freshwater systems: to avoid native predators, prey organisms typically display particular behaviours (reviewed by [6], [7]) that may be inappropriate or ineffective when dealing with novel invaders. For example, novel predator cues may fail to activate an apt defence response [8]–[13]. Consequently, introduced predators pose a more serious threat to prey species than native predators [14]. Parallel to their deleterious effects, species invasions have also provided, especially in isolated habitats such as islands or remote aquatic systems, ‘natural experiments’ that could be used to better understand relationships between predators and their prey [1], [15]. Nevertheless, the specific behavioural interactions between natives and invaders are usually only poorly known.

A notable example of the above-mentioned isolated habitats is provided by Nicaraguan crater lakes, which are inhabited by several species of the Midas cichlid complex (within the genus *Amphilophus*: [16], [17]). These fish are distinguished by pronounced trophic polymorphisms [18]–[21], striking colour morphs [22]–[24] and highly complex behaviours [25]–[27]. Indeed, Midas cichlids have become one of the most prominent systems for study of biological diversification [17], [28]–[30]. This is especially true for the arrow cichlid, from hereon *Amphilophus zaliosus*, which is endemic to Nicaraguan crater lake Apoyo, and has most likely evolved within the lake, providing one of the best cases of sympatric speciation [17], [31], [32]. Observational evidence [31], catching success during previous studies [21], [32], [33], and a population genetic analysis over a 16 year period [28] suggest that, until recently, *A*. *zaliosus* were common in the lake. Furthermore, a field survey indicated that over the period as recent as 1997–2005, on average 5% of all reproductively active fish of the Midas cichlid species complex in Lake Apoyo were *A*. *zaliosus* [34]. However, our observation from the breeding season of 2007–2008 suggest much lower occurrence (see below), which is in accordance with the decision of the IUCN Red List of Threatened Species to recently (in 2010) list *A*. *zaliosus* as a critically endangered species [35].

The definitive reasons for the decline of *A*. *zaliosus* are currently not known. It has been suggested that invasive species, especially introduced African cichlids (tilapia; *Oreochromis*), may compete for food or breeding space, or carry diseases to which *A*. *zaliosus* are susceptible [36], [37]. However, no tilapias were encountered during the present study and these are thought to have become very rare in Apoyo. In contrast, the most common and most abundant invasive fish species in the lake is the predatory bigmouth sleeper (*Gobiomorus dormitor*), which was introduced into the lake in 1991 by local fishermen [38], [39]. The species, however, occurs naturally in many of the western Nicaraguan lakes, including crater lake Xiloá [38], [39]. In this study we assessed behavioural interactions between brood-tending *A*. *zaliosus* and bigmouth sleepers intruding their territories. The same assessment was also conducted for another species within the Midas cichlid complex, *A*. *sagittae*, that has coevolved in sympatry with bigmouth sleepers in Xiloá. This natural experiment allowed us to investigate the consequences of novel predation pressure in terms of adjustment of brood defence behaviours and to assess the potential of the new invader to contribute to the decline of *A*. *zaliosus*.

## Methods

### Study sites and focal species

**Figure 1:**
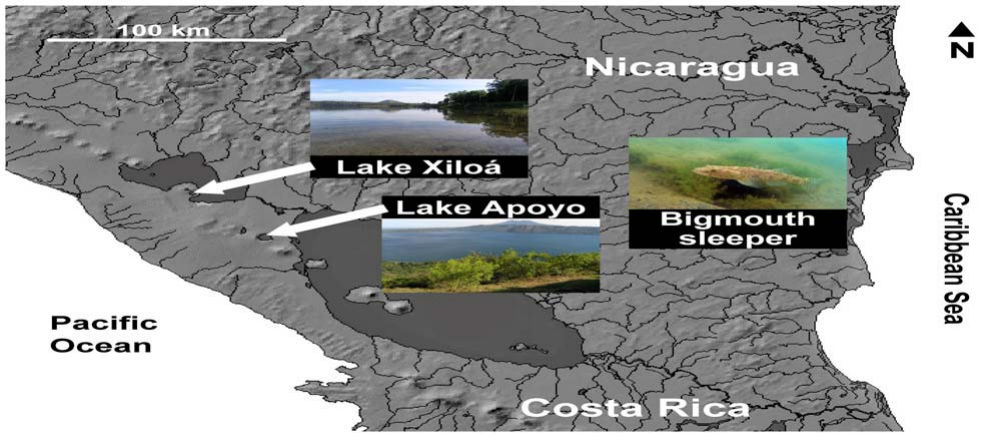
Lake Apoyo and Lake Xiloá. Apoyo and Xiloá are crater lakes, i.e. volcano calderas filled with water.

Nicaraguan crater lakes Apoyo and Xiloá (Figure 1) have received particular attention from evolutionary biologists because of their interesting assemblage of endemic cichlid species and their exceptionally clear water that allows underwater observations [24], [31]–[33], [40]–[42]. Like other members of the Midas cichlid species complex, *A*. *zaliosus* (the arrow cichlid) and *A*. *sagittae*, endemics to Apoyo and Xiloá, respectively, form stationary breeding territories for the duration of a reproductive cycle (authors' personal observations, see also [26], [42]–[44]). During each breeding cycle, these fish exhibit extensive parental care (usually biparental), which continues for a month after the juveniles have started to swim, with the juveniles reaching the total length of ca. 3 cm in that time ([26], [43], [44], authors' personal observations). The two species resemble each other phenotypically and ecologically more than they resemble any other species within the Midas cichlid complex: both species have elongated bodies, reach total length of approximately 20 cm, are silvery-coloured fish that develop dark breeding coloration, exhibit partly piscivorous feeding habits, and outside the breeding season, have a more pelagic habit than other species within the group [16], [17], [31], [32], [40], [42]. The two species seem to have speciated within their two respective lakes [24], [32], [40], and hence are not, despite the extensive phenotypic similarity, each other's closest living relatives.

The main predator of cichlid fry in the two above-mentioned crater lakes, the bigmouth sleeper (*Gobiomorus dormitor*) (Figure 1), is an eleotrid fish with a wide distribution in tropical and subtropical brackish and freshwater systems in the New World [45]. Bigmouth sleepers are ambush predators that become increasingly piscivorous as they increase in size [46] and commonly reach a length of 25 cm [39]. Stomach content analyses, as well as our personal observations, indicate that, in both lakes, the bigmouth sleeper is the most important single predator on cichlid broods, with cichlid fry being a major component of the diet of the species [39]. Potential brood predators, other than bigmouth sleepers, are also similar in the two lakes: this category comprises of conspecifics, other species within the Midas cichlid complex, and juveniles of cichlids of the genus *Parachromis* (authors' personal observations). In addition to these, five smaller cichlid species, which might opportunistically predate on fry of other cichlids, inhabit Lake Xiloá ([47], [48], authors' personal observations). In both lakes, brood predation by invertebrates or non-cichlid fish species is also conceivable, but probably not significant, since we did not witness any non-cichlid species (other than bigmouth sleepers) within the reaction distance of the territory-guarding parents during this study.

### General study procedures

We hypothesised that parental defences of brood guarding cichlids may be less efficient towards bigmouth sleepers when this prominent predator is introduced than when the brood guarding species has coevolved with it. Correspondingly, to examine the responsiveness of parental fish to novel brood predation, we compared aggressive responses by parental *A*. *zaliosus* in relation to (i) bigmouth sleepers and (ii) the rest of the fish predators. Furthermore, we conducted the same comparison with *A*. *sagittae* in crater lake Xiloá. These comparisons were conducted between December 2007 and January 2008 using SCUBA, and involved approximately 33 hours of underwater data-gathering with a further 45 dive hours devoted to localising breeding pairs (mainly in Apoyo). Furthermore, an additional survey during good water visibility conditions in Xiloá (see below) was conducted for assessing *A*. *sagittae*, between December 2010 and January 2011, and involved approximately 25 dive hours. The study was carried out under research permits from the Ministerio del Ambiente y los Recursos Naturales (MARENA), Nicaragua (Permit numbers: DGRNB-IC-006-2007 and No. 026/-11007/DGAPw).

### Parental behaviour in 2007–2008

After a breeding territory of *A*. *zaliosus* (in Lake Apoyo, Figure 1) or *A*. *sagittae* (in Lake Xiloá, Figure 1) was located, the observer maintained a distance of approximately two metres from it. The date, water depth, horizontal visibility estimate, habitat/substratum type, and approximate total lengths of the parents and offspring were recorded. Offspring size estimations were initially based on our personal observations on the change of offspring appearance over time, and these ‘age’ assessments were later transformed to absolute estimations of size. The adult length estimates, in turn, were calibrated by occasionally catching individuals of these and closely related species (see [42]). The calibration revealed a relatively consistent 10% over-estimation of the total length, which was subsequently reduced from the original approximations. These linear corrections did not affect the outcomes of the statistical tests. In other respects, our protocol for assessing brood defence behaviour of the territory holders closely followed those applied by Lehtonen et al. [27], [49]. Specifically, after a habituation period of three minutes (with the observer lying immobile on the bottom), the activities of the parental fish were recorded for 15 minutes. We classified each act of brood defence behaviour by the parent fish according to one of two categories: acts of ‘display aggression’ were behavioural threats such as flared fins and gills, with gradual or no movement towards the fish invading the territory. ‘Mobile aggression’ (equivalent to ‘attacks’ + ‘chases’ in [49]) involved pronounced and usually rapid movement towards the intruder. For each act of aggressive brood defence, we also noted the reaction distance (as an approximation of territory size), estimated as the distance between the centre of the brood and the invading fish at the time of the response. After the observation period was finished, the territory was marked with a yellow stone to prevent pseudoreplication. Because of a very low occurrence of actual predation (fry mortality) within any given observation period, we were not able to collect systematic data on predation success. In the few cases where we did observe successful predation events on cichlid offspring, the successful predator was always a bigmouth sleeper.

Territorial responses were assessed for 29 of the 30 *A*. *zaliosus* broods that were encountered in the course of this study. The territories of *A*. *sagittae* (*n* = 26) included in the study were chosen at random within the subset of the encountered broods that were at free-swimming stage but estimated not having been swimming for more than three weeks (equivalent to total length of ca. 2¼ cm). This subset was chosen to match brood ages of the two species. *Amphilophus zaliosus* breeding pairs encountered during this study favoured a substratum consisting of a mix of rock and sand (*n* = 23 out of 30) and usually associated with a covering of *Chara* green algae. The remainder of *A*. *zaliosus* breeding pairs occurred on bare rock and stones of various sizes (7/30). *Amphilophus sagittae* territories were occasionally found in both pure sand (here: 1/26), and pure rock (1/26) habitats, but most commonly the species was encountered in the mixed habitat (24/26).

During the 15-minute observation periods, 25 *A*. *zaliosus* and 18 *A*. *sagittae* broods were approached by both bigmouth sleepers as well as other potential offspring predators (i.e. any individuals of cichlid fish, especially of genera *Amphilophus* and *Parachromis*, large enough to eat fry), allowing paired comparison between the two predator groups (i.e. bigmouth sleepers versus ‘other predators’). Correspondingly, the rest of the breeding territories were excluded from most analyses because these territories were approached by only a single predator type during the observation period (approaches by bigmouth sleepers only: *A*. *zaliosus n* = 1, *A*. *sagittae n* = 0; approaches by ‘other predators’ only: *A*. *zaliosus n* = 3, *A*. *sagittae n* = 8). Furthermore, some of the broods we observed were defended by only a solitary female (see [42], [49]). We nevertheless included these in our data analyses because the observed pattern in reaction distances remained qualitatively the same even (i) when single females were excluded from the analyses, (ii) when only the behaviour of the female parent was analysed, or (iii) when female status (paired vs. solitary) was added as a covariate. Note, however, that we did not have a sample of male size for the broods that were defended by solitary females. We estimated the range of underwater vertical visibility during the study period as 2–5 metres in both lakes. However, during the dives which included brood defence behaviour measurements, the estimated horizontal visibility was higher in Apoyo (4.28±0.14 m) [mean ± standard error] than Xiloá (2.90±0.12 m).

Parametric statistical tests were only applied when their criteria were met. To compare the distances of intruders from the centre of the territory at the time territory holders reacted aggressively to them, we included both cichlid species in the same repeated-measures analysis of variance (RM-ANOVA). Specifically, the averaged values of reaction distances toward bigmouth sleepers and other offspring predators per territory comprised the paired (‘repeated’) dependent variable and the species of the territory holder (*A*. *zaliosus* or *A*. *sagittae*) was used as a factor. It is possible that in some cases territory holders reacted to the same intruder more than once. However, due to the high abundance of the predators in both lakes, it is very unlikely that any individuals were observed at multiple territories. Furthermore, as each data point consists of the averaged distances over one territory, the data-points can be regarded as independent of each other. The comparisons of proportions (aggression type or predator type) were conducted on arcsine (square root) transformed data. Because the means and standard errors of proportional data were also calculated after the transformation, we subsequently needed to reverse-transform these back to proportions (and further to percentage), which explains our asymmetrical standard error estimates.

### *Amphilophus sagittae* parental behaviour in 2010–2011

To address the possibility that the lower visibility in Xiloá than Apoyo in 2007–2008 could have biased our results, we have included an additional survey on *A*. *sagittae* during the breeding season of 2010–2011, when water in Xiloá was clearer (estimated horizontal visibility: 3.96±0.14 m). *Amphilophus zaliosus* were not observed during that breeding season because logistic challenges allowed only a very limited dive time in Apoyo, during which no *A*. *zaliosus* breeding pairs were encountered (although immature individuals were sighted). The procedure for assessing parental responses was the same as above, with the following two exceptions: brood defence of only biparentally guarded broods were assessed and the observation period was 10 min, instead of 15 min per territory. In total, parental behaviour of 26 brood tending pairs were measured, and 19 of these (males: 21.3±0.3 cm, females: 18.3±0.3 cm) were approached by both types of predators, allowing a paired comparison (paired *t*–test).

## Results

### 2007–2008

**Figure 2:**
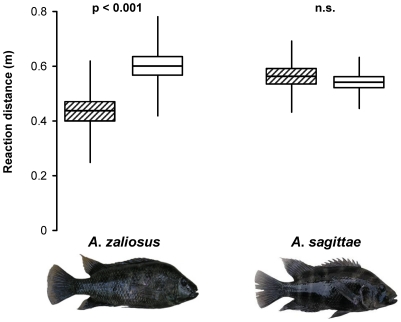
Distances from the centre of the territory to which potential offspring predators had advanced before territory holders reacted to them aggressively. This comparative data was collected during the 2007–2008 breeding season. Hatched boxes show reaction distances towards bigmouth sleepers and white boxes are for other predators. Central vertical lines indicate means, margins of the boxes are for standard errors of the means, and whiskers indicate standard deviations. Sample sizes (both predator groups) are 25 and 18 for *A*. *zaliosus* and *A*. *sagittae* breeding territories, respectively.

There was a significant interaction between the species of brood tenders (*A*. *zaliosus* or *A*. *sagittae*) and the type of predator (bigmouth sleepers or other predators) (RM-ANOVA, predator type × species interaction, *F*_1,41_ = 13.8, *p* = 0.001). We therefore proceeded to analyse the two cichlid species separately and found that parent fish responded aggressively towards bigmouth sleepers only when these had approached closer than other potential brood predators were allowed to approach in *A*. *zaliosus* (paired *t* – test, *t*_24_ = 4.62, *p*<0.001; Figure 2) but not in *A*. *sagittae* (paired *t* – test, *t*_17_ = 0.680, *p* = 0.51; Figure 2). Furthermore, the proportional use of display aggression vs. mobile aggression was not dependent on the predator type in *A*. *zaliosus* (paired *t* – test, *t*_24_ = 0.668, *p* = 0.51) or *A*. *sagittae*: (paired *t* – test, *t*_17_ = 1.09, *p* = 0.29). The total rate of aggressive acts towards potential brood predators did not significantly differ between the two species (*A*. *zaliosus*: 1.86±0.16 1/min, *n* = 29); *A. sagittae* (1.56±0.13 1/min, *n* = 26; two-sample *t* – test, *t_53_* = 1.48, *p* = 0.15). Furthermore, the two species did not significantly differ in the proportion of aggressive behaviours that they directed towards bigmouth sleepers vs. other potential brood predators (proportion directed towards bigmouth sleepers in *A*. *zaliosus*: 37.9 +7.2/−6.9%, *n* = 29, and in *A. sagittae*: 24.1 +7.6/−6.9%, *n* = 26; Mann-Whitney *U* – test, *U* = 301, *p* = 0.20).

The *A*. *zaliosus* and *A*. *sagittae* individuals in 2007–2008 were approximately of the same size, were breeding at similar depths and defended juveniles of the same estimated size (Table 1). However, sizes of juveniles were similar only because they were matched in order to eliminate any age bias that could potentially result in differences in brood defence behaviour.

**Table 1:** Comparison of the observed breeding territories and their residents.

Species	Male length (cm)	Female length (cm)	Territory depth (m)	Fry length (cm)
*A*. *zaliosus*	23.6±0.5	20.1±0.4	8.5±0.4	1.18±0.05
*A*. *sagittae*	22.1±0.5	19.4±0.4	9.3±0.4	1.28±0.08
				
**Comparison**	*t*_25_ = 1.98, *p* = 0.06	*T*_41_ = 1.20, *p* = 0.24	*t*_25_ = 1.45, *p* = 0.16	*t*_25_ = 1.10, *p* = 0.29

The values [mean ± standard error] for the two species, in the upper part, are compared using two-sample *t* – tests in the lower part.

### 2010–2011

*Amphilophus sagittae* parents (in Xiloá) reacted from a longer distance to bigmouth sleepers than other predators (paired *t* – test, *t*_18_ = 6.47, *p*<0.001). As in 2007–2008, the proportional use of display aggression vs. mobile aggression was not dependent on the predator type (paired *t* – test, *t*_18_ = 0.245, *p* = 0.81).

## Discussion

Compared with native fish predators, the non-native bigmouth sleepers were able to approach the broods of *A*. *zaliosus* closer before they were chased away. However, this was not the case for brood tending *A*. *sagittae*, which naturally occur in sympatry with bigmouth sleepers. Indeed, in water clarity conditions similar to those prevailing in Apoyo (breeding season 2010–2011), parental *A*. *sagittae* actually reacted to bigmouth sleepers from a greater distance as compared to the rest of potential predators of their offspring. By advancing more closely to *A*. *zaliosus* broods than other potential predators are able to approach, the introduced ambush predator can be expected to be particularly effective in capturing juveniles (see [50]). This is especially true since both types of predators try to capture juveniles by dashing after them, but only after having been able to approach close enough (authors' personal observations). Why, then, did *A*. *zaliosus* in Lake Apoyo allow bigmouth sleepers to advance so closely?

In communities where predators and prey have coexisted for long periods, prey have evolved behaviours or morphologies that enable them to cope in an adaptive way with the predators they may encounter [7]. In contrast, species facing novel predators may, at least initially, lack an appropriate response behaviour [8]. Our results suggest that *A*. *zaliosus*, which evolved in Lake Apoyo (which has existed no longer than approx. 20000 years, see [17]) in the absence of bigmouth sleepers, may not be able to detect these ambush predators as effectively from a distance as they detect native predators, or *A*. *sagittae* parents detect bigmouth sleepers in Lake Xiloá (which formed approximately 6000 years ago, see [17]). Alternatively, *A*. *zaliosus* simply do not regard these novel predators to be a severe threat to their offspring compared to the native predators with which they have coevolved. In any case, our results demonstrate an inappropriate behavioural response to an introduced predator, a mechanism which may have resulted in many freshwater organisms to be particularly sensitive to introduced predators (*sensu* [8]).

Some alternative hypotheses, besides ‘evolutionary inexperience’ of parental *A*. *zaliosus*, could also help to explain why bigmouth sleepers can approach so closely to *A*. *zaliosus* broods. We first considered the possibility that bigmouth sleepers, which are somewhat different from cichlid predators in their morphology and swimming pattern, were more efficient than other predators in approaching Midas cichlid broods because they were introduced into conditions of high water clarity in Apoyo. There are, however, several lines of evidence suggesting that this hypothesis is unlikely to explain our results. Most importantly, at the time water clarity was high also in Xiloá (2010–2011), brood-tending parents reacted to bigmouth sleepers from a farther distance as compared to other predators. In other words, if the difference in water clarity affected the relative reaction distances, it should have made our results more conservative. However, our other results suggest that the difference in water clarity did not have a major effect on the ability of the parents to deal with brood predators. Specifically, we did not notice any differences in the behaviour of bigmouth sleepers or other predators in the two lakes, the total rates of aggressive behaviours were similar for the two cichlid species, and we did not find any difference in the ability of ‘other predators’ to approach the broods in the two lakes.

Besides differences in water clarity, we also considered the following additional alternative hypotheses: (i) behaviour of bigmouth sleepers may be plastic, leading to a higher predation efficiency in Apoyo than Xiloá, (ii) cichlid parents could have fewer opportunities for phenotypic habituation (see [51]) in Apoyo, or they need to rely on predator species recognition only in that lake, and (iii) non-bigmouth sleeper predators might be less efficient at approaching cichlid broods in Lake Apoyo. We propose, however, that each of these options is likely to have, at best, only a minor role in explaining our results for the following reasons. First, we did not notice any differences in the behaviour of bigmouth sleepers in the two lakes [relevant with respect to point (i) above]. Second, the total reaction rates towards bigmouth sleepers, as well as towards all intruders combined, were similar for the two cichlid species (in 2007–2008) [(i) and (ii)]. Third, 90% (26/29) and 69% (18/26) of the territories examined in 2007–2008 in Apoyo and Xiloá, respectively, were approached by bigmouth sleepers within the 15-minute observation period [(ii)]. Fourth, a great majority of territory intruders are potential predators on the brood in *both* lakes [(ii)]. Fifth, we did not find any difference in the ability of ‘non-*Gobiomorus* predators’ to approach the broods in the two lakes [(iii)]. Finally, the exclusion of the few opportunistic brood predators (smaller cichlid species) that are not shared by the two lakes does not qualitatively change our results on reaction distances [(iii)].

It seems that the selection regime on breeding *A*. *zaliosus* has drastically changed within the last twenty years, i.e. after introduction of the bigmouth sleeper. We found that currently ca. 40% of all aggressive responses of *A*. *zaliosus* parents were directed towards the introduced predator, which is in line with the current, very high density of this novel predator in the lake ([39], authors' personal observations). Furthermore, the estimated length for none of the *A*. *zaliosus* juveniles, encountered during the two month study covering most of the 2007–2008 breeding season, was above 2 cm. This is important because Midas cichlid offspring become independent of their parents about a month after starting to swim when they have reached the length of 3 cm ([44]; authors' personal observations on both species considered here) and juveniles of 2 cm or smaller are not large enough to disperse. This pattern suggests an extremely low rate of successful reproduction attempts at the population level. In contrast, we constantly encountered *A*. *sagittae* broods with an approximate size of 3 cm (or age of at least four weeks), suggesting that in this species broods had a higher rate of survival. Our field evidence, in this regard, is consistent with the concern of IUCN Red List: during the peak breeding season in Lake Apoyo, more than 40 dives with an average duration exceeding one hour were needed to localise 30 breeding territories of *A*. *zaliosus*, while thousands of territories of other Midas cichlids were still encountered. This indicates much lower incidence of reproductively active *A*. *zaliosus* than just a few years earlier (see introduction for more details). Similarly, non-breeding *A*. *zaliosus* were encountered relatively rarely. However, we note that although the species has apparently declined simultaneously with the spread of bigmouth sleepers, we do not currently have data to link these patterns directly to each other.

The majority of the cichlids observed reproducing in Apoyo during this study were *A*. *astorquii* [41] individuals breeding in dense colonies amidst *Chara* green alga beds in relatively shallow water (3–8 meters; TKL personal observations). This breeding strategy may work better against the impact of bigmouth sleepers than the one of *A*. *zaliosus* (see [48]). Intriguingly, 22 of the 30 *A*. *zaliosus* breeding territories encountered during this study were located on *Chara* beds near, or even within, *A*. *astorquii* colonies, although the species has previously been reported to use exclusively rocky caves for breeding [31]. This shift may indicate that the selection of a new type of breeding habitat can be a quicker response to novel predation pressure than adjustment of brood defence behaviour. For example, at the time of introduction of bigmouth sleepers, *A*. *zaliosus* may have had a higher potential for phenotypically plastic breeding habitat choice than for an optimal set of defence behaviours towards a novel predator (see [12], [52]).

Here we have shown that arrow cichlids, *A*. *zaliosus*, reacted to the bigmouth sleepers only when the introduced predator had already advanced significantly closer to the brood than native predators were allowed to approach. By advancing so close, the novel ambush predator should be expected to be particularly effective in capturing juveniles. Our study is the first to suggest that inappropriate brood defence behaviour due to (evolutionary) naiveté towards novel predators may have resulted in sensitivity of some (or many) freshwater organisms towards introduced predators. We regard this to be important because understanding the exact interactions by which introduced species affect native inhabitants can help to predict and avert the negative impacts of species invasions and more aptly direct conservation measures.
